# Case report: adult onset diabetes with partial pancreatic agenesis and congenital heart disease due to a de novo GATA6 mutation

**DOI:** 10.1186/s12881-020-01012-2

**Published:** 2020-04-03

**Authors:** Begona Sanchez-Lechuga, Muhammad Saqlain, Nicholas Ng, Kevin Colclough, Conor Woods, Maria Byrne

**Affiliations:** 1 Department of Diabetes & Endocrinology, Mater Misericordiae University Hospital, Dublin 7, Ireland; 2 Department of Diabetes & Endocrinology, Tallaght University Hospital, Dublin 24, Ireland; 3 Department of Molecular Genetics, Royal Devon & Exeter NHS Foundation Trust, Exeter, UK

**Keywords:** Adult-onset, Diabetes, GATA6, Pancreatic agenesis, Treatment

## Abstract

**Background:**

Mutations in GATA6 are the most frequent cause of pancreatic agenesis. Most cases present with neonatal diabetes mellitus.

**Case presentation:**

The case was a female born after an uncomplicated pregnancy and delivery in a non-consanguineous family (3.59 kg, 70th percentile). Severe cardiac malformations were diagnosed at two and a half months old. No hyperglycaemic episodes were recorded in the neonatal period. Diabetes was diagnosed at 21 years due to the detection of incidental glycosuria. She had a low but detectable C-peptide level at diagnosis. Anti-GAD and Islet-cell antibodies were negative and she failed oral hypoglycaemic therapy and was started on insulin. Abdominal MRI revealed the absence of most of the neck, body, and tail of pancreas with normal pancreas elastase levels. Criteria for type 1 or type 2 diabetes were not fulfilled, therefore a next generation sequencing (NGS) panel was performed. A novel heterozygous pathogenic GATA6 mutation (p.Tyr235Ter) was identified. The GATA6 variant was not detected in her parents, implying that the mutation had arisen de novo in the proband.

**Conclusion:**

Rarely GATA6 mutations can cause adult onset diabetes. This report highlights the importance of screening the GATA6 gene in patients with adult-onset diabetes, congenital cardiac defects and pancreatic agenesis with no first-degree family history of diabetes. It also emphasizes the importance of genetic counselling in these patients as future offspring will be at risk of inheriting the variant and developing GATA6 anomalies.

## Background

The formation of beta-cells during embryonic development is regulated by several transcription factors (TFs) that activate specific genes. Many of these TFs are also important in pancreas function during adult life [1]. The GATA family represents a group of conserved zinc finger TFs involved in development and differentiation of eukaryotic organisms. Two subgroups have been described in vertebrates, including the hematopoietic GATA1/2/3 and cardiac groups GATA 4/5/6 [2]. This last group is expressed in tissues of endodermal and mesodermal origin including gut, lung, heart and pancreas [3]. Mutations in GATA6 are the most frequent cause of pancreatic agenesis [4, 5]. It is also associated with neonatal diabetes mellitus (NDM), congenital cardiac malformations and other extrapancreatic features such as biliary tract defects, gut abnormalities [4, 6] and significant neurocognitive deficit [5, 7]. The most common pancreatic feature is diabetes occuring in 98% of cases. The vast majority of cases present with NDM, but a small proportion develop adolescent or adult onset diabetes [7].. This report describes a 21 year old female diagnosed with adult-onset diabetes associated with congenital heart defects and pancreatic agenesis associated with a de novo heterozygous mutation in the GATA6 gene.

## Case presentation

**Fig. 1:**
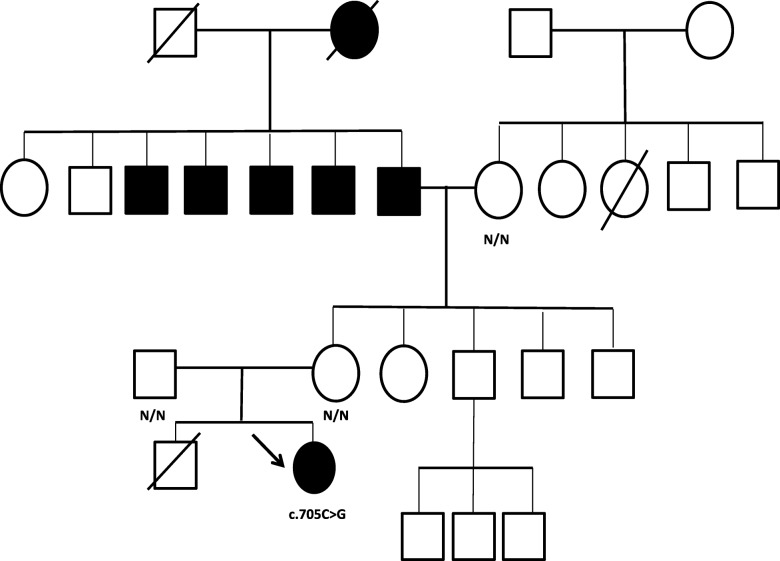
Family pedigree. An arrow points to the *index case*. Squares represent male family members, and circles represent female family members. Filled black symbols represent people with diabetes. Genotype is shown underneath each symbol. N/N denotes no mutation identified

She was born at 40 weeks gestation after an uncomplicated pregnancy and delivery in a non-consanguineous family. Her birth weight was 3.59 kg (70th percentile). She was diagnosed with a severe cardiac malformation (subaortic ventricular septal defect, small atrial septal defect and moderate patent ductus arteriosus) at two and a half months old. At 5 months old, coartation repair, PDA ligation and pulmonary artery banding were performed. Some months later she had the VSD repaired and pulmonary artery deep banding. She had mild residual coarctation, bicuspid aortic valve with mild aortic stenosis and mild aortic incompetence. She had normal growth with adult height of 165 cm. She also had scoliosis and hypertension (on losartan 100 mg). She completed secondary school and works as a health care assistant. No hyperglycaemic episodes were recorded in the neonatal period. At 21 years old, incidental glycosuria was detected and diagnosis of diabetes was confirmed. Baseline fasting bloods showed glucose 9 mmol/l, Insulin 19.7 mUI/l (2–25) and C-peptide 2.8 μgr/l (1.1–4.1). HbA1c was 61 mmol/mol, and anti-GAD and Islet cell antibodies were negative. Pituitary and thyroid function were normal. On examination, BP was 102/72 mmHg, HR 74 bpm and BMI was 24.3 kg/m2. She has no features of insulin resistance: no striae, acanthosis nigricans or skin tags. She had a 2/6 aortic ejection systolic murmur with a 2/4 early diastolic murmur. Significant scoliosis was noted. She was started on metformin, but this was discontinued due to gastrointestinal side effects, and gliclazide MR 30 mg once daily was started. There was a positive family history of diabetes shown in Fig. 1. There was no family history of cardiac defects or pancreatic agenesis. Her mother reported a still birth at 7 months before the index *case* was born (unknown cause of death).

**Fig. 2:**
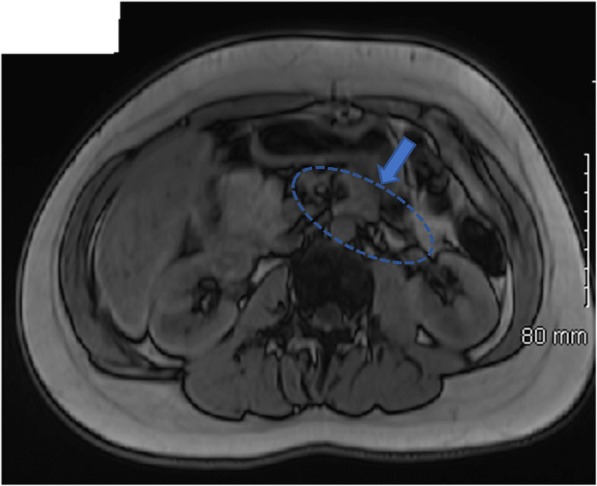
Abdominal MRI axial and coronal image revealing the absence of most of the neck, body and tail of pancreas

She was referred with atypical diabetes to the maturity onset diabetes of the young (MODY) clinic in 2017 with a HbA1c of 59 mmol/l on gliclazide which was increased to 60 mg daily. An oral glucose test was performed (14 months after diagnosis) off treatment for 2 days. Fasting glucose was 10.3 mmol/l and her 2-h post load glucose was 18.4 mmol/l; fasting C-peptide was 484 pmol/l and 2 h post load was 512 pmol/l. Subsequently, gliclazide MR was titrated to 120 mg daily. Three months later blood glucose level was 32 mmol/l (HbA1c 95 mmol/mol) and gliclazide was discontinued. Multiple dose injection therapy was started. Her current HbA1c is 45 mmol/l on 25 units of insulin daily. Incidentally, during investigations for other disorders, an abdominal magnetic resonance imaging revealed the absence of most of the neck, body and tail of pancreas (Fig. 2). Pancreas elastase was > 500 μg/g (normal > 200 μg/g). She had no evidence of background retinopathy and her microalbuminuria to creatinine ratio was normal.

**Fig. 3:**
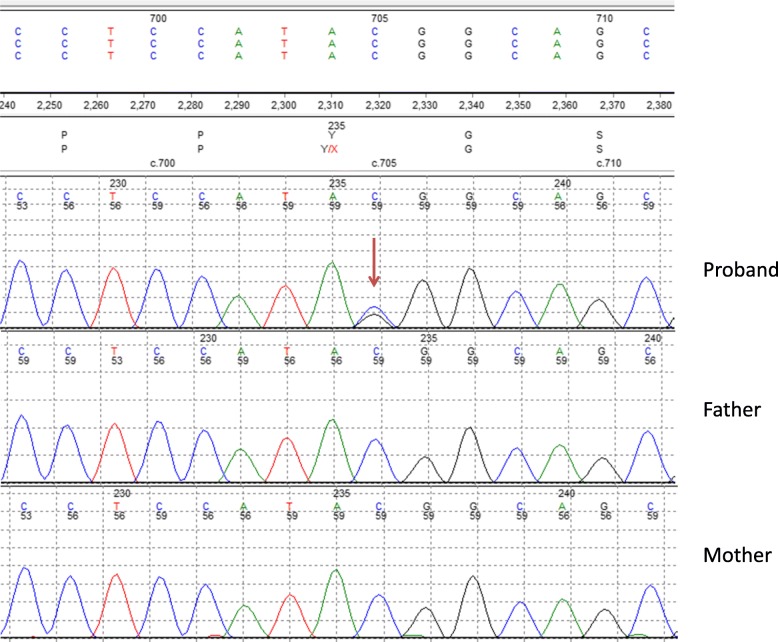
Sanger sequencing electropherogram of *GATA6* exon 2 showing the heterozygous C > G single base substitution at nucleotide position 705 (c.705C > G) resulting the nonsense variant (p.Tyr235Ter) in the proband. The variant is absent in electropherograms from mother and father. Sanger sequencing is able to detect low level somatic mosaicism to a level of approximately 5–10%

Patient did not meet criteria for either type 1 or type 2 diabetes and was categorized as “Other diabetes”. A mutation in hepatocyte nuclear factor 1B was suspected initially given her diabetes profile and pancreatic agenesis. A renal and pelvic ultrasound scan was performed and no abnormality was detected. Targeted NGS analysis of 34 known monogenic diabetes genes identified a novel heterozygous pathogenic *GATA6* nonsense variant, (p.Tyr235Ter) (NM_005257.5:c.705C > G) in exon 2. This variant is classified as pathogenic class 5 according to the AMCG guidelines [8]. The variant generates a premature termination codon in exon 2 and the mRNA transcripts are predicted to undergo nonsense mediated decay, resulting in haploinsufficiency for GATA6 (Fig. 3).

The *GATA6* variant was not detected in peripheral blood samples from her non diabetic parents (mother HbA1c 28 mmol/mol, FPG 3.9 mmol/l and father HbA1c 37 mmol/mol, FPG 5.5 mmol/l) and maternal grandmother (Fig. 1), implying that the mutation had arisen de novo in the proband. Low level somatic mosaicism in both parents was excluded to a level of ≥5%. The possibility of germline mosaicism could not be excluded.

## Discussion and conclusion

This report describes a 21 year old female diagnosed with adult-onset diabetes associated with atrial septal defect, subaortic ventricular septal defect, patent ductus arteriosus and pancreatic agenesis and a novel de novo heterozygous mutation (p.Tyr235Ter) in the *GATA6* gene.

Pancreatic agenesis is a rare disorder resulting in NDM associated with exocrine pancreatic insufficiency [9]. The commonest cause of pancreatic agenesis or hypoplasia is heterozygous inactivating mutations in *GATA6* [5, 7, 10].. The prevalence of *GATA6* mutations is unclear but a few studies have shown that mutations account for at least 3% of NDM and 54% of patients with pancreatic agenesis [5]. The most common phenotypes are congenital cardiac defects, particularly outflow tract malformations such as atrial and ventricular septal defects or tetralogy of Fallot [4]. *GATA6* mutations can also lead to several other phenotypes: congenital biliary tract anomalies (17%), gut developmental disorders (21%), neurocognitive (38%) and additional endocrine abnormalities (24%, hypothyroidism/hypopituitarism) [4, 5, 7].

Pancreatic agenesis or hypoplasia can cause permanent NDM and has been associated with mutations in TFs important for β cell and pancreatic development., GATA6 is the most common of these mutations [11].. The severity of diabetes varies among family members, ranging from NDM with only a remnant of pancreatic tissue to adult-onset diabetes associated with dorsal agenesis of the pancreas [12–14]. The mechanism whereby GATA6 mutations result in associated clinical phenotypes is still not completely understood.. The mutation (p.Tyr235Ter) in the GATA6 protein occurs in the transcriptional activation domain which is full of pathogenic variants. There are multiple nonsense mutations close by and even a missense mutation on the same amino acid. A recent publication by Škorić-Milosavljević et al. [15], highlights the broad mutational and clinical spectrum and lack of genotype-phenotype correlation in patients with GATA6 mutations. Their study showed that 58% of probands had de novo mutations and these patients had more frequently anomalies of intracardiac connections and the great arteries and they were more likely to have hypothyroidism when compared to patients who had inherited a mutation. Our patient did not have hypothyroidism. The phenotypic spectrum suggests the existence of modifier genes. This is a syndromic disorder, and therefore by its nature will have a variable clinical phenotype and penetrance just as we see with other dominantly inherited syndromic forms of diabetes such as HNF1B where there is no genotype-phenotype correlation [16].

De Franco et al. [5] found that while investigating 171 patients with neonatal diabetes, four parents with heterozygous GATA6 mutations (c.1136-2A > G, p.(Tyr323*), p.(Thr346fs) p.(Gly469Glu), were diagnosed with adolescent or adult-onset diabetes (12–46 years). A retrospective study for pancreatic donors with diabetes showed a 16 years old girl diagnosed with GATA6 mutation p.(Arg456Cys) and adolescent-onset diabetes [14]. It remains unclear if environmental factors or the different genetic background of other genes can interact with GATA6 [13]. Reviewing the literature, a de novo mutation in GATA6 p.(Arg456His) was recently shown to be associated with young adult-onset diabetes type 1 at age 22 in a patient with pancreatic hypoplasia [15]. The other two patients with adolescent or adult-onset diabetes (c.1504_1505delAA) were detected due to investigations for family members with neonatal diabetes [13]. The mutations do not represent a separate class. Summarizing, it has been described a total of 3 adolescents and 5 adults onset diabetes. Interestingly, our patient did not show growth retardation or failure to thrive. In addition, her faecal elastase levels were normal and she only developed diabetes at age 21 years. This is consistent with a previous report of phenotypic heterogeneity ranging from pancreatic agenesis to adult onset diabetes with no evidence of exocrine pancreatic insufficiency [5]. Her findings are consistent with dorsal pancreatic agenesis as previously described in patients with adolescent or adult onset diabetes with GATA6 mutations [12, 13]. Interestingly, she failed sulphonylurea therapy and required insulin within 13 months of diagnosis. There is not an extensive description in the literature of the diabetic treatment of GATA6 patients who develop adolescent or adult onset diabetes. In fact there are only 8 cases described and 6 were treated with insulin, one with metformin and one treatment unknown [5, 13–15].

It should be emphasized that the use of targeted NGS for clinical diagnostic testing will increase the number of patients with a confirmed diagnosis of monogenic diabetes. NGS technology enables the potential for simultaneous analysis of all the known disease genes in a single assay at a similar cost to analysing a few genes by Sanger sequencing [17]. Despite the fact that the majority of *GATA6* mutations occur de novo, a small number of dominantly inherited cases have been reported [12]. Yorifuji et al. [13] reported the case of a family with a dominantly inherited mutation and neonatal complications resulting in death in 3 of 4 pregnancies. A genetic diagnosis is important since it defines the diagnostic subtype, determines the most appropriate treatment and informs the sibling recurrence risk or risk of GATA6 associated anomalies in offspring.

In conclusion, this report describes a heterozygous novel *GATA6* mutation (p.Tyr235Ter) resulting in the clinical picture of congenital heart defects, dorsal pancreas agenesis and adult-onset diabetes in a patient with no first-degree family history of diabetes. It emphasizes the importance of screening the *GATA6* gene in young patients with diabetes and congenital cardiac defects. It also highlights the importance of genetic counselling in these patients as future offspring will therefore be at risk of inheriting the variant and possibly developing a much more severe phenotype that could include pancreatic agenesis and congenital heart defects.

## Data Availability

All data is available in this manuscript, this case report did not require any analysis. The raw sequencing data has been submitted to NCBI’s sequence read archive (SRA). The accession number is SRX7816321 and the link to the data submission.

## Abbreviations

NGS: Next generation sequencing
TFs: Transcription factors
NDM: Neonatal diabetes mellitus
MODY: Maturity onset diabetes of the young
